# Children’s Diet during the Early Stages of the Nutritional Transition. The Foundlings in the Hospital of Valencia (Spain), 1852–1931

**DOI:** 10.3390/ijerph182211999

**Published:** 2021-11-15

**Authors:** Francisco J. Medina-Albaladejo, Salvador Calatayud

**Affiliations:** 1 Department of Economic Analysis, Faculty of Economics, Universitat de València, Avinguda dels Tarongers s/n, 46022 València, Spain; 2 Institut Interuniversitari López Piñero and Department of Economic Analysis, Faculty of Economics, Universitat de València, Avinguda dels Tarongers s/n, 46022 València, Spain; salvador.calatayud@uv.es

**Keywords:** living standards, nutritional transition, children nutrition, nutritional balance, hospital diets, inequality, Spain, 19th and 20th centuries

## Abstract

The nutritional transition brought about profound changes in the nutrition of the European population in the 19th and 20th centuries. The predominant consumption of cereals gave way to kilocalorie-, protein-, vitamin- and mineral-rich diets that involved a greater intake of animal products. However, not all population groups underwent this transition at the same pace; socio-economic conditions, sex and age led to important inequalities. This article uses institutional sources to analyse the nutrition of children during the early stages of the nutritional transition and to compare it with that of other age groups (adult psychiatric patients). The study examines the average diets and nutritional balance of foundlings in the Hospital General de Valencia from 1852 to 1931. The main conclusion of the study is that, throughout the period under study, foundlings were exposed to a poor, traditional diet, characterized by structural deficits and imbalances. This may have affected their physical growth, health and biological wellbeing in adulthood, and demonstrates that the nutritional transition was anything but a homogeneous process.

## 1. Introduction

Over the last century, an intense historiographical debate has discussed the evolution of the living standards of the working class during the process of industrialization, involving the examination of demographic, economic, anthropometric and wellbeing indicators. Within this broad historiographical trend, increasing attention has been paid to the evolution of alimentary patterns and changes in diet structures. The growing consumption of kilocalories, proteins and animal fats, which partially replaced the traditional diet based on cereals and other vegetal foodstuffs, between the mid-19th century and the first half of the 20th century, is a process known as the nutritional transition. In Spain, this process began later than in other European countries, owing to a belated economic and industrial development. This slowed down a series of processes that the specialised literature has identified as key for the nutritional transition, including increased income levels, improved production and distribution of animal products, scientific–technical advances and urbanisation, among others [1,2,3,4,5,6].

The nutritional transition and its kilocalorie-, protein- vitamin- and mineral-rich diets improved the nutrition of the Spanish and European populations. This had a direct effect on mortality rates, life expectancy, height and health [7]. However, it is reasonable to ask if all population groups enjoyed the advantages of the nutritional transition equally, and thus to determine the effect of inequality factors such as socio-economic status, sex and age [8,9].

Nutritional needs change with age (Life Course Perspective) and are particularly sensitive to income, so it is not unreasonable to think of children as being particularly vulnerable to nutritional deficits, especially when they depend on family units within which food resources are unevenly distributed. Studies based on the resource dilution model suggest the possible impact of family size on the allocation of childcare duties on children [10]. In terms of food consumption, attempts have been made to validate the model based on differences in stature [11,12]. However, historiography has paid little attention to child nutrition, with a few exceptions, such as Crawford’s study of Irish workhouse diets in the 19th century [13]. The children hosted by this institution ranged in age from 9 to 15 years, and their diets were based on potatoes and bread; Hawkins and Tanner examined the alimentary practices of a British children’s hospital in the 19th century and the early 20th century, emphasizing the therapeutic value assigned to nutrition in these institutions to care for urban working-class children who arrived to hospital in poor physical and nutritional condition [14]. More recently, Scholliers studied the nutrition of children (aged 2–14) in the 19th century based on the regulations of two hospitals in Brussels. This author argues that no general trends about child nutrition in Europe can be established, although there is solid evidence that it followed a similar path to adult diets (but they were always comparatively poorer). Scholliers concludes that child nutrition deteriorated during the first half of the 19th century, to improve rapidly in the second half of the century, especially in the decades that followed the First World War [15,16]. Studies which are not based on institutional diets agree that child nutrition during this period was highly deficient [17,18].

The primary aim of this article is to assess child nutrition in the context of the early stages of the nutritional transition through the analysis of institutional sources, which can provide valuable evidence for the issue at hand, as well as to outline some of the factors that affected this change in the diets. Specifically, we have examined the diets of foundlings in the Hospital General de Valencia from 1852 to 1931 in order to establish their main characteristics and evolution over time, and estimate some of the possible causes of these diet changes and their nutritional balance in comparison with other hospital diets. The composition of the diet takes into account foodstuffs consumed, kilocalorie content and nutritional balance, and is compared with the Recommended Dietary Allowances (RDA) [19], which are especially important for child development.

The paper is organized as follows. Following the Introduction, the Materials and methods sections describes in detail the sources and methodologies used. The Results section analyses the evidence, which is contextualized and interpreted in the Discussion section. Finally, the paper closes with a Conclusions section.

## 2. Materials and Methods

Hospital diets can be used to reconstruct effectively consumed individual rations and track their changes over time within a given population group. This evidence enables us to go beyond national averages based on apparent consumption. Although the records used correspond to a very specific population group (hospital patients), which arguably undermines their representativeness, many examples attest that hospital and other institutional diets in Europe were often similar to those that the population consumed outside these institutions (prisons, workhouses, mental hospitals, orphanages…) [9,13,15,20,21,22]. Since hospitals mostly hosted members of low-income population groups below the poverty line, patient diets could be regarded as similar to those consumed by these social groups in their everyday life. It is, therefore, reasonable to assume that, during the period under examination, hospital and domestic diets would not differ too much. However, few studies have used hospital and institutional diets in Europe or Spain to examine nutrition in the 19th and 20th centuries [9,23,24].

The Hospital General de Valencia’s archives keep on record the food entering the pantries and kitchens, as well as the amount of food served to each patient. The document “Estado del consumo de los víveres que constituyen la ración en las diferentes clases de albergados” enabled us to establish the daily diet of foundlings. This document records the total quantities of food being released by the hospital pantry daily, as well as the number of patients fed and the number of days that each patient was in hospital. The information was recorded by the nun in charge of running the pantry, under the supervision of the pantry official. The period under study has been divided into six sub-periods, which broadly represent what are widely regarded as major trends in the early stages of the Spanish nutritional transition [4]: 1852–1854, 1866–1868, 1899–1901, 1909–1911, 1921–1923 and 1929–1931. For the periods 1852–1854 and 1866–1868, we used three of these daily records (on the 10th, 20th and 30th day of the month) for each month, in order to create a homogenous sample. For 1899–1901, 1909–1911, 1921–1923 and 1929–1931 the daily data were added by months, and we used this aggregate information. Using 1852 as start date for the series responds to the incompleteness of earlier records, owing to the habit of patients and visitors of bringing food, which complemented or replaced the institutional diet. The regulations published in 1850 forbade this practice, so from this date onwards there is greater certainty that the food served by the hospital constituted the patients’ only source of nutrition [25]. The uneven time interval between these periods responds to the limitations of the source (no evidence for some years, or it was not disaggregated). Foundlings began taking solid food at the age of 20 months, and remained in the institution until the age of 7, unless they were adopted. From that age, they passed to another institution, the Casa de la Misericordia, which cared for people of all ages in severe poverty. Table 1 presents the data corresponding to the number of days that foundlings spent in hospital during the period under consideration. In the 19th century, the hospital fed approximately 50 foundlings per day, which amounted to more or less 5% of the hospital’s patients. This percentage decreased gradually but firmly in the following decades, as the number of abandoned children went down. In terms of gender, the proportions were fairly even, although the number of girls was slightly higher (Archivo de la Diputación Provincial de Valencia (hereafter, ADPV), sección Hospital, II-6/C-2, C-4, legs. 17, 18, 49, 50, 51, *Estadística mensual de expósitos*, 1866, 1867, 1868, 1899, 1900, 1901).

The methodology used to process the data and calculate the nutritional balance of diets is as follows. First, the amounts of food consumed by the foundlings in one year was added up and divided by the number of hospitalization periods (one hospital day) recorded by the source; this yields a precise per capita consumption figure. The foodstuffs served varied little during the period under study, the main changes having to do with the amounts served. These foodstuffs were rice, white bread (made with wheat but little bran) and brown bread (also made with wheat, but with more bran, which made the bread darker in color and cheaper in price. Brown bread ceased to be served in the hospital in the 1920s, when it was replaced by white bread) [26] (p. 450); chickpeas; potatoes; meat (veal); wine; olive oil; and chocolate. To this, we must add milk from the hospital’s cows, which was accounted for differently than the other foodstuffs; as the amount that foundlings took was not recorded, a ration identical to that of another animal-based product, meat, was assumed. Therefore, these figures are merely an estimate. The calculations also leave out foodstuffs that were acquired daily in the local market, the records for which do not specify amounts or to whom they were served: vegetables, spices, onions, garlic and sardines. In any case, these products must have been of little importance overall as, according to hospital accounts, their purchase never exceeded 4.5% of the hospital’s food budget (ADPV, sección Hospital, II.1.14, V-2.3. *Presupuestos de gastos*).

Second, the nutritional profile of the diets was established, using a widely used methodology [3,23,27]. After subtracting the percentage of non-edible elements from the total quantity of each product consumed (raw), using the Base de Datos Española de Composición de Alimentos (Spanish food composition database; BEDCA; www.bedca.net/bdpub/index.php (accessed on 15 September 2021)) transformation coefficients per 100 g were calculated using the same source. This database was used by previous studies [8,23,28]. One problem of this methodology comes from applying recent known compositions to past foodstuffs. Although some studies suggest that differences in composition exist [29,30], the lack of past nutritional analysis makes the use of current criteria inevitable, even if this leads to bias which, at any rate, does not seem to be too significant. This methodology is widely used in studies dealing with nutritional intake. Recent years have witnessed an interesting debate around this issue [31,32,33]. This method results in homogenous series for the whole period under study, enabling valid comparisons that are impossible to base on unprocessed hospital records. The data were organized as follows: (a) Composition of the diet by product, with daily intake and relative weight in terms of kilocalorie consumption; (b) Relative distribution of macronutrients: proteins, fats and carbohydrates. Maximum biological value protein contents were based on the values published by FAO [34]; (c) The proportion of animal-based proteins in total protein intake; (d) Nutritional balance, comparing the diets’ kilocalorie and nutrient content with RDA values, considering proteins as the primary macronutrient and all micronutrients (vitamins and minerals). Especially important micronutrients for child development were singled out: vitamins A, D and folic acid (vitamin B9), and minerals such as calcium, iron and zinc. According to the specialized literature, these are the most important micronutrients for an adequate physical and intellectual development and the satisfactory operation of the immune system [35,36].

The calculation of RDA of the foundlings in each of the sub-periods was based on the food composition tables published by Moreiras et al. and population censuses [19]. As noted, foundlings were given solid food at the Hospital between the ages of 20 months and 7 years. These tables provide separate RDA for age brackets 1–3, 4–5 and 6–9, which provides highly accurate estimates. In addition, a weighted average, based on the sex and age structure of the infant population of the province of Valencia (according to censuses), was calculated. This provides for more reliable and accurate results than in previous publications about the same population group [9], which used the RDA calculated by Cussó et al. for the Spanish infant population as a whole (0–14 years) [8], which were, therefore, a looser fit for the group analysed here, which is children between the ages of 20 months and 7 years.

Finally, the nutritional balance of the foundlings’ diet has been compared with that of well-off psychiatric patients and poor psychiatric patients. These two groups were defined by the hospital regulations, and they constituted a significant proportion of the hospital’s population, accounting for over 40% of total hospital days (Table 1). These groups include adults (over 20 years of age) in all age groups [9]. Generally, men were more numerous than women, amounting to over 60% of psychiatric patients overall between 1860 and 1923, and to 70% of well-off psychiatric patients (ADPV, sección Hospital, III-4/C-1, legs. 6, 8, 9, 10, 17, 18, 19, 28, 29, 30, *Estadística mensual del manicomio provincial de Valencia*, 1860, 1879, 1880, 1881, 1901, 1902, 1903, 1921, 1922, 1923; ADPV, sección Hospital, III-4/C-2, C-8, C-9, legs. 34, 35, 36, 64, 65, 66, *Estadística diaria del manicomio provincial de Valencia*, l895, 1896, 1903, 1921, 1922, 1923). In the late 1920s, the hospital ceased distinguishing between well-off and poor psychiatric patients, and their number decreased sharply, probably owing to the construction of a new psychiatric hospital.

## 3. Results

Table 2 presents the amounts consumed by foundlings in each of the sub-periods under study. In the 19th century, products from the traditional Mediterranean diet are clearly predominant: bread, white and, especially, brown, which was regarded as inferior; other cereals (rice); legumes (chickpeas); meat (veal); and olive oil. With the crystallization of the changes brought about by the early stages of the nutritional transition in the early decades of the 20th century, the diet became progressively richer and more varied. The substantial reduction in the number of abandoned children may have also been an important factor in this (Table 1). White bread entirely replaced brown bread in the 1920s, and the daily amounts served increased to half a kilogram in 1929–1931. This change is explained by the greater popularity of white bread and the hospital’s improved financial position in the 20th century. Meat consumption also increased, after half a century of gradual decline, because by the mid-19th century the hospital’s services had deteriorated after half a century of recurrent crises and loss of income, especially when the sale of the mortmains had liquidated some of its assets [37] (p. 42) [38] (p. 351). By 1929–1931, meat consumption had not yet gone back to the levels reached in the first and second subphases. However, a gradual increase in meat consumption is attested from 1899–1901. The hospital’s financial difficulties led to deteriorating services and alimentary deficits [39] (p. 190). It must be taken into account that meat was among the most expensive food items [9] (p. 1038). New foodstuffs in the diet also included milk, chocolate and potatoes. However, the foundlings had no access to products that were served to other patients, such as noodles, beans, salted cod, pork fat, poultry and eggs. This was set out in the institution’s regulations, which established the type of food to be served each population group (ADPV, sección Hospital, *Reglamento para el suministro de raciones y sistema pensionario*, 1872).

Especially significant is the absence of fruit, vegetables and fresh fish which, as we shall see shortly, resulted in significant deficits in the intake of vitamins and other micronutrients in all the hospital’s diets, but especially in those consumed by foundlings. As noted, these products were used in the hospital only sparingly. These low consumption levels are further attested by other sources. For instance, Giral Pereira’s study on nutrition in Spain estimates that the average daily diet (no distinction is made by age, gender or income levels) included only 69 g of vegetables, 103 g of fruit, and 11 g of fresh fish [40] (p. 314). Concerning institutional diets, such as those consumed by army recruits and jail inmates, these products were entirely absent [40] (pp. 330, 332–333). Other primary and secondary sources confirm this circumstance. For instance, the average daily diet in Barcelona (another city, such as Valencia, in the Mediterranean coast), barely included 2 g of fruit, 100 g of vegetables, and 22 g of fresh fish in 1902 [41] (p. 527). Furthermore, the diet of an industrial labourer for that same year was entirely lacking in these products, except for potatoes [42] (pp. 58–59), and the same can be said about the average diet served in the Hospital Clínico de Barcelona in 1909 [41]. Similar situations have been attested in other countries [14,15,17,18].

The kilocalorie intake of these infant diets ranged from 1500 to 2200 kilocalories during the period under study, as illustrated by Table 3. In the 19th century, this variable changed little, but in the opening decades of the 20th century, kilocalorie intake increased substantially, largely as a result of increases in bread consumption. Hospital diets met the foundlings’ energy requirements, as we shall see further below, but the structure of the diet was predominantly based on cereals. During the period under study, bread and rice accounted for between 65% and 70% of total energy intake, and only in 1929–1931 did this drop to 60%. The consumption of animal-based products, mostly meat, remained fairly constant, between 15% and 23%. Between 1909–1911 and 1921–1923 there was a period of stagnation related to the elevated inflation caused by the First World War. In this period, the price of meat in the city of Valencia went up by 106% and that of wheat flour by 84%, which may have driven down the presence of these products in the diet in 1921–1923, breaking the earlier trend [43]. A cost of living index for the period 1908–1920 suggests an increase of 150% [44] (pp. 101-102) [45] (p. 178) [46] (pp. 161–168) [47] (p. 185). Therefore, the evolution of prices could have had an impact on foundlings’ diets, in the same way that the drop of real wages limited access to food to wage labourers. This relationship, however, does not seem so clear in other periods [9].

Until maximum biological value proteins were introduced into the diet with milk towards the end of the period under study, bread and meat were the foundlings’ main protein source. The main sources of fat were meat and, especially, olive oil, the consumption of which remained constant, although always at levels below the recommended amounts. In contrast, the consumption of carbohydrates (in the form of cereals) was not only predominant throughout, but actually tended to increase over time. Between 70% and 75% of the kilocalories consumed came from carbohydrates, significantly above the 50–60% recommended range. This imbalanced distribution of macronutrients was a problem shared by all poor groups in the hospital, but not so much by certain groups of privileged patients, as we shall see shortly.

**Figure 1:**
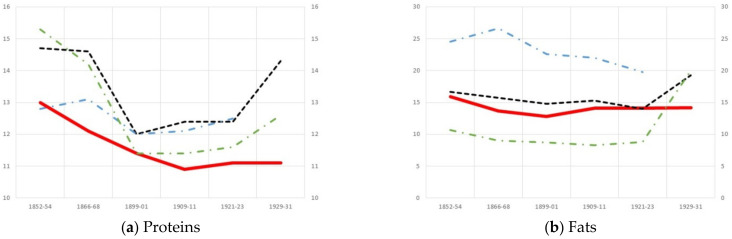

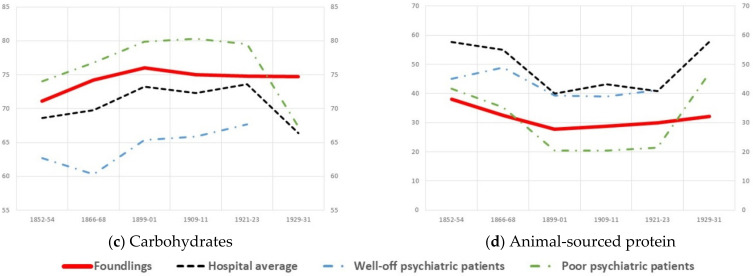
Distribution of macronutrients (in percentage) (**a**–**c**) and percentage of animal proteins (**d**) in the average daily diet of foundlings and other population groups in the Hospital General de Valencia, 1852–1931 (%). Source: see Table 1, Table 2 and Table 3. Data of well-off psychiatric patients and poor psychiatric patients: [9].

If these results are compared with those yielded by other patient groups or with the hospital’s averages, the predominance of carbohydrates and the secondary importance of proteins (especially animal based) and fats in the diet of foundlings is confirmed. Figure 1 illustrates the distribution, in percentages, of macronutrients and the role played by animal proteins among foundlings, the hospital average (including patients and staff), and among two specific groups: well-off psychiatric patients and poor psychiatric patients. Figure 1a shows that the lowest relative weight of proteins is found in foundling diets, even below the poorest groups of psychiatric patients. In fact, this relative weight gradually decreased until 1910 with the consumption of meat, the source of maximum biological value proteins. The trend was reversed in 1910, but at a much slower pace among foundlings than in the hospital’s averages or among poor adult psychiatric patients. Figure 1d confirms that the foundlings’ consumption of maximum biological value proteins was similar to that of poor adult psychiatric patients and much below that of wealthy psychiatric patients and hospital averages. In the 20th century, this indicator improved owing to the consumption of milk, but at a much slower pace than among poor psychiatric patients and the hospital average.

The relative weight of carbohydrates in the diet remained fairly constant during the period under study, and above hospital averages (Figure 1c), although it was lower than among poor adult psychiatric patients. Fat consumption was close to the average (Figure 1b), and considerably above that of poor adult psychiatric patients, owing to the elevated consumption of olive oil. The weight of carbohydrates in the average diet and in the diet of poor adult psychiatric patients dropped dramatically towards the end of the period under study, but this decrease was much less sharp among foundlings.

Was this a balanced diet that met the RDA of foundlings in the context of the change brought about by the nutritional transition? Table 4 shows that the foundlings’ diet met their minimum energy and protein requirements throughout the period under study. During the 19th century, the diet was above 90% of the RDA calculated for a population ranging in age between 20 months and 7 years. In the 20th century, the increase in the amounts of traditional foodstuffs consumed and the introduction of new ones significantly improved the values associated with this indicator, and by 1929–1931 the diet was more than 30% above the estimated RDA. Protein requirements were also amply met during the whole period under consideration.

However, when it comes to micronutrients, severe deficits emerge, which reflect the imbalance of infant diets during the period under study. This could have a significant impact on the growth, health and development of these children at a later age, although it is fair to say that gross nutrition improved significantly during the final stages of the period under analysis. As shown in Table 4, in the mid-19th century the diets presented severe shortcomings, especially in terms of vitamins and some minerals such as calcium and iodine.

Vitamins A and D were in short supply throughout the period under study. The former is essential for growth and the development of the immune system, and is mostly found in eggs, dairy and vegetables. The short allowance of these products in the hospital food meant that this micronutrient was virtually absent from the diet. During the 20th century, the intake of vitamin A increased with milk, although to levels that were still far below the RDA. The lack of vitamin D, which plays an essential role in the mineralization of the bones by improving the absorption of calcium and phosphorus, and thus in avoiding rickets, was even more severe and persistent, owing to the absence of such products as fish and eggs from the foundlings’ diets. Deficiencies in vitamins A and D were a widespread problem in Spain as a whole [3], but the hospital records suggest that among the foundlings this must have been especially serious, affecting their growth.

With other vitamins, the deficits were not as concerning, although their intake remained far below the RDA; for instance, vitamin E, owing to olive oil consumption, and vitamin C, the intake of which increased substantially with the addition of potatoes to the diet in 1929–1931. Similarly, the intake of thiamine, riboflavin and vitamin B6 increased substantially with the consumption of bread and milk, reaching the RDA by the end of the period. Finally, the intake of such vitamins as niacin, folic acid and B12 consistently met the RDA during the whole period under study, because they are present in significant quantities in bread and meat.

Concerning calcium, a mineral that plays a crucial role in growth and the formation of the bones, intake levels, which largely relied on bread, were low until 1929–1931, as were the consumption of zinc and iodine. The former is basic for growth and the operation of the immune system, and is generally present in significant quantities in high-protein foodstuffs, such as meat, legumes and cereals. The main sources of zinc for the foundlings were bread and meat, and intake levels, such as those of iodine, improved significantly with the incorporation of milk to the hospital diets in the opening decades of the 20th century. Conversely, the infant diets contained substantial quantities of iron, which helps to transport the oxygen required by cell metabolism; its absence, therefore, can cause anaemia and lead some organs to malfunction. This micronutrient is found in animal-based foods, legumes and vegetables, and its main sources in the hospital diets were bread, meat and chickpeas. Other minerals consumed in sufficient quantities were phosphorus (bread, meat and milk); magnesium (bread, chickpeas and chocolate); and, especially, selenium (bread).

**Figure 2:**
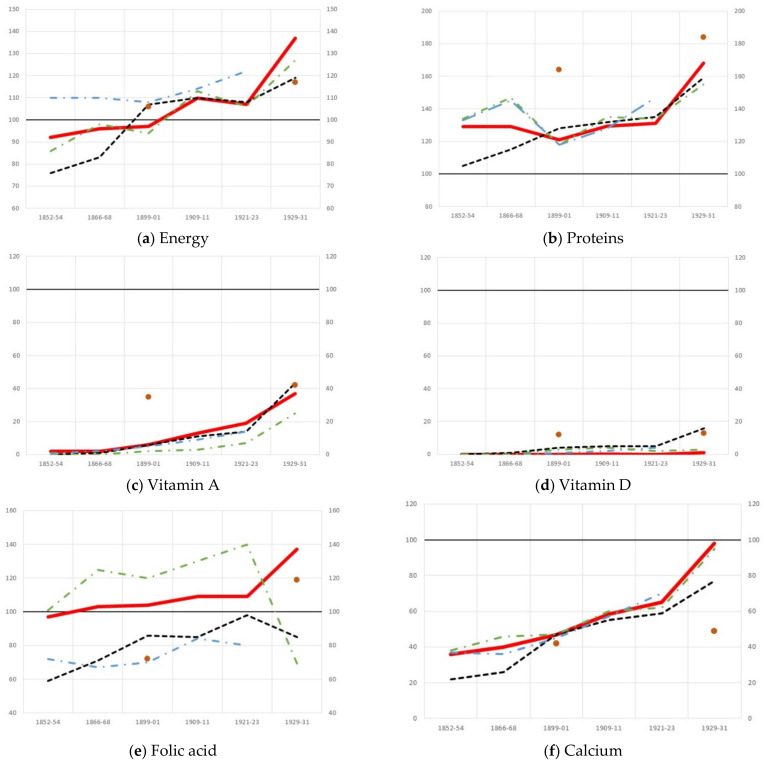

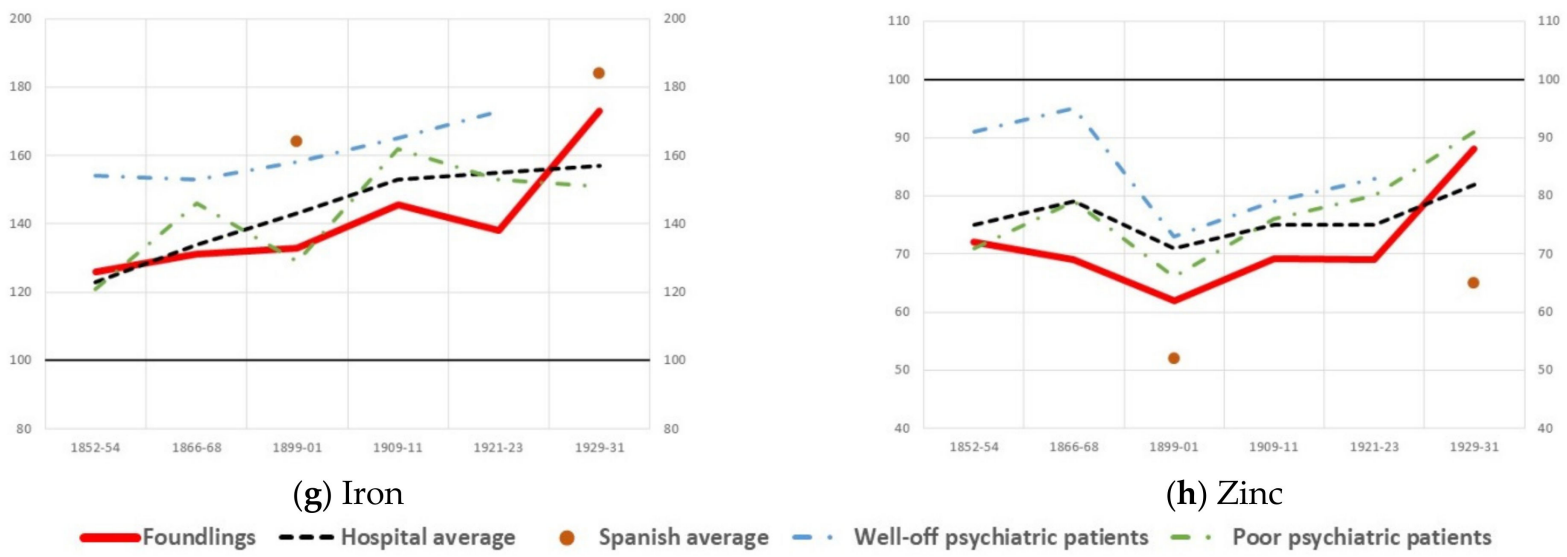
Contribution of energy and nutrients with respect to the Recommended Dietary Allowances (RDA = 100) in the average daily diet of foundlings and other population groups in the Hospital General de Valencia, 1852–1931. Source: see Table 1, Table 2 and Table 3. Data of Spanish average, well-off psychiatric patients and poor psychiatric patients: [9].

These imbalances in the foundlings’ diets were also found in other patient groups, but some of them were more severe among the foundlings. As shown by Figure 2a,b, the upward trend in energy and protein intake applies to all groups. Deficiencies in the consumption of vitamins A and D were widespread, not only in the hospital, but in the Spanish population as a whole. On the other hand, the presence of folic acid in the foundlings’ diet evolved positively owing to the frequent consumption of legumes. Similarly, increases in calcium consumption were common to all hospital diets, especially among foundlings as a result of the consumption of milk. It is worth noting that, towards the end of the period under study, milk consumption in the hospital was well above that of the population as a whole, in which the consumption of milk made headway but slowly, and at first only among very specific social groups [15,51,52]. The results also show that foundlings consumed comparatively less iron and zinc than other hospital groups, as their intake levels remained below average.

## 4. Discussion

The previous section shows that, in general, infant diets in the Hospital General de Valencia were similar to those of other hospital groups in the period under study, especially that of unprivileged adult psychiatric patients, whose diet was always much poorer than those of wealthier psychiatric patients. In the 1920s, hospital diets improved in general, but that of foundlings did so less than the rest, as they rarely incorporated the new animal-based foods that were becoming more common in the context of the early stages of the nutritional transition.

This conclusion agrees with some of the ideas that the literature has put forth, but other arguments can be challenged. According to Scholliers, infant and adult diets in Brussel’s hospitals in the late 19th and early 20th centuries were essentially the same, except in terms of ration size, which broadly agrees with our conclusions, with some caveats [15,16]. Foundlings in Valencia were systematically denied certain products, some of which played an important role in the nutritional transition, such as pork fat, poultry and eggs.

The diets presented by Scholliers for Brussels seem to have been less caloric but more balanced than those of the Valencian foundlings; their profile corresponds to a more advanced stage of the nutritional transition. However, it has to be taken into account that Scholliers used only hospital regulations, which are no guarantee that foundlings in Brussels were in fact receiving the foodstuffs and quantities conveyed by the source. In Hospital Saint-Jean, the diet of children aged 7 to 10 amounted to 1400 kcal in 1877, a similar amount to the 1548 kcal given to Valencian foundlings aged 20 months to 7 years in 1866–1868. However, foundlings in Brussels were given 200 g of meat, milk and butter, while Valencian foundlings received a more traditional diet, with less meat (117 g), no vegetables, milk or butter, and nearly 400 g of bread. In the Hospital of Sant Pierre, in 1884, foundlings were allowed to eat as much as they wished of some foodstuffs at given times, including white coffee, bread, rice pudding or buttered bread. In addition, they received 200 g of vegetables and 150 g of meat. As a result, the protein and vitamin intake was much higher in Belgian hospitals than in Valencia [15]. Concerning the periodisation of our results, the second half of the 19th century witnessed a moderate increase in kilocalorie intake, but a deterioration of the nutritional balance, as a result of the increase in the amount of bread and the decrease in that of meat in the diet, before the trend was reversed in the three opening decades of the 20th century. It is also observed that, when diets improved, that of foundlings did so at a slower pace than the rest, even those of the most unprivileged patient groups. All of this broadly agrees with the periodisation established by Scholliers, who pointed to a clear improvement in the diet after the First World War, although he also argued that diets had been gradually improving from the mid-19th century onwards [15,16]. The latter phenomenon is not attested in Valencia, no doubt as a result of the financial difficulties faced by the Hospital General during the 19th century, where, in addition, the number of foundlings cared for was significantly higher than in the early decades of the 20th century (Table 1).

In the 19th and 20th centuries, Spanish liberal institutions financed social welfare services with difficulties [53] (p, 944) [54] (p. 366), and these were partially delegated to private initiatives. Historically, the finances of the Hospital General of Valencia were based on the fees paid by those who could afford them, donations and the rents derived from the agricultural land owned by the institution. As the latter was largely lost after the sale of the mortmains in 1854, the hospital began receiving public funds (Ayuntamiento and Diputación Provincial de Valencia), but these were insufficient, and large donations by the city’s upper classes [37,38]. On several occasions in the second half of the 19th century, the prices paid by the hospital for basic foodstuffs increased significantly, for instance flour in the 1870s (63% more than in the previous decade) and meat in the 1880s (27.4%). This led to an increase in the real cost of foundlings’ diets, and this could have driven down the consumption of meat, which accounted for nearly half of the money spent on feeding the foundlings [9] (pp. 1033,1038–1039). In fact, the hospital’s expenses in food, which amounted to approximately 90% of the institution’s budget at that time, substantially decreased from 1870 and did not reach earlier levels until the 1890s. In addition, in the 1880s, additional funds had to be added to the annual budget several times, to address: *“The substantial price increase in several of the products consumed at this hospital, especially meat, wheat, poultry and eggs”*. These extra expenses amounted to approximately 9% of the institution’s total food expenses (ADPV, sección Hospital, II.1.14, V-2.3. *Presupuestos de gastos*).

Therefore, our results reinforce the traditional notion that financial concerns carried more weight than medical and nutritional criteria, and that institutions that were not financially sound presented their patients with inadequate food [13,15,55]. Scholliers and Maes et al. concluded that children suffered greater nutritional deficiencies than adults [15,16,21], and the diet of foundlings in Valencia seems to agree with this. However, Scholliers’s conclusion that infant diets improved faster than those of adults is not applicable to our case study [15]. Said improvements were triggered in Europe by a more favorable financial context for the hospital institutions and the emergence of new medical theories concerning the nutrition of children in the late 19th century [14,15].

Concerning quality and nutritional balance, the diet of Valencian foundlings provided enough kilocalories and was rich in proteins, but presented severe nutritional imbalances during the whole period under study. The main reason for this was the insufficient presence of certain vitamins and calcium in the diet. Diets analysed by other studies present similar results. In his seminal article about the nutrition of the English working class in the 19th century, Oddy (1970) pointed out the nutritional deficiencies faced by children, whose diet, in which vegetables were almost entirely lacking, was based on bread and potatoes [17]. Crawford reached similar conclusions in his study of infant diets in Irish workhouses during the 19th century, as did Davin in his study about working class households in early 20th century London, in which hunger was not rare [13,18]. Scholliers flagged significant imbalances in hospital diets in Brussels [15,16]. The most significant deficits affected kilocalorie intake (only in the second half of the 19th century), vitamins, iron and calcium. Hawkins and Tanner pointed out that malnutrition was a common problem among the children admitted to the Great Ormond Street Hospital for Sick Children, who suffered a wide array of related illnesses (rickets, weakness and marasmus), that fruit was virtually absent from the diet and that vegetables were served only twice a week [14]. Even the most optimistic studies about working class diets, such as Clayton and Rowbotham’s and Gazeley and Newell’s, admit that infant malnutrition was a common problem [27,56].

These nutritional shortcomings could contribute to explain the high mortality rates of foundlings admitted to the Hospital General de Valencia, which in the mid-19th century were between 50% and 75%. In this period, the hospital received approximately 800 foundlings per year [57] (p. 163). The mortality rate had not gone down by 1900, when it reached 77.2% [58] (p. 337). This problem, which has been paid substantial historiographical attention, was widespread in Spanish and European charitable institutions. Financial difficulties negatively affected the nutrition of older foundlings, but also of breastfeeding children, as paying the internal and external wet nurses’ wages became difficult. Other factors, such as the poor hygienic conditions and transport difficulties, may have also played a part [59,60].

## 5. Conclusions

This article has analysed changes in the diet of foundlings in the Hospital General de Valencia in the context of the early stages of the nutritional transition. This has enabled us to accurately outline the nutritional deficiencies faced by infants, in line with other studies about infant nutrition in Western Europe. The data presented give a quantitative perspective of an issue that is often formulated qualitatively. The use of hitherto underexploited sources has made it possible to determine the nutritional profile of the diets and, therefore, their differences with those provided to adult psychiatric patients. Foundlings were among the most unprivileged social groups, and their diet was based on traditional foods and generally poor in vitamins, so that by the 1920s they had barely begun to feel the positive effects of the nutritional transition. These dietary differences were especially significant concerning animal-based foodstuffs, which were more expensive.

All this emphasizes that the nutritional transition was anything but a homogeneous process, and that it did not involve a generalized change in diet structures. During the whole period under study, the diet of foundlings was characterized by structural imbalances and deficits, although things improved in the opening decades of the 20th century. Valencian foundlings faced important deficits in vitamins and some minerals, owing to the excessive predominance of cereals in their diet and the insufficient quantities of animal products, fruits and vegetables consumed. This could affect their growth, health and biological wellbeing later in life.

These results can also contribute to a more fact-based approach to the evolution of sanitary conditions during the whole life cycle. As reliable primary sources are scarce, no studies have yet addressed the relationship between infant nutrition and growth in later stages from a historical perspective, a pending challenge for historical research. However, anthropometric history has put forward some evidence upon which some tentative hypotheses can be based. According to the literature, the height of Mediterranean Spanish 21-year-old adults barely increased during the second half of the 19th century, growing at a faster pace from the late 19th century and, especially, the 1920s onwards [61,62]. This trend is particularly pronounced among low-income social groups [62,63]. Many environmental factors affect height, but nutrition is one of the most important among them. Was the evolution in the height of army recruits in the second half of the 19th century related to the deficits in infant nutrition reflected in hospital diets, and to the general improvement of the diet in the 20th century? Historiography must address these questions in the future by digging out reliable primary sources that enable us to reconstruct past diets, especially among the most vulnerable social groups.

## Figures and Tables

**Table 1:** Total number of days spent by foundlings and other population groups in the Hospital General de Valencia, 1852–1931.

	1852–1854 *	1866–1868 *	1899–1901	1909–1911	1921–1923	1929–1931
Foundlings						
Total number of days	43,962	61,938	36,372	15,628	5211	9690
% of total hospital days	4.8	5.7	2.7	0.9	0.4	1.1
Number of patients cared for per day	40	57	33	14	5	9
Well-off psychiatric patients						
Total number of days	30,893	22,995	29,896	41,504	74,889	-
% of total hospital days	3.4	2.1	2.2	2.5	5.1	-
Number of patients cared for per day	28	21	27	38	68	-
Poor psychiatric patients						
Total number of days	377,795	512,572	471,528	515,844	548,200	28,356
% of total hospital days	41	47.4	35.4	31.2	37.2	3.1
Number of patients cared for per day	345	468	431	471	501	26

Notes: * the data for the periods 1852–1854 and 1866–1868 are estimates based on 3-day samples per month and year, as explained above. The data collected for 108 days for each 3-year period were used to calculate the average number of patients per day. The total number of hospital days in each period was obtained by multiplying this figure by 365 days and three years. This method is reliable, as the examination of the primary sources confirmed that the average number of foundlings cared for during the period 1866–1868 is very similar to the estimate yielded by this methodology (56). Source: ADPV, sección Hospital, II,2.1, V-7.2/2-3,33, V-7.7/7,10, *Estado del consumo de los víveres que constituyen la ración en las diferentes clases de albergados de este Hospital Provincial*, 1852, 1853, 1854,1866, 1867, 1868, 1899, 1900, 1901, 1909, 1910, 1911, 1921, 1922, 1923, 1929, 1930, 1931; ADPV, sección Hospital, II-6/C-1, leg. 8, *Estadística diaria de expósitos*, 1866, 1867; ADPV, sección Hospital, II-3/4, *Libro de partes desde 1868 a 1873*, 1868.

**Table 2:** Average daily diet of foundlings in the Hospital General de Valencia, 1852–1931.

Product	1852–1854	1866–1868	1899–1901	1909–1911	1921–1923	1929–1931
Brown bread (g)	227.6	280.5	290.1	303.1	0	0
White bread (g)	120.3	118.1	114.7	119.4	419.6	503.1
Rice (g)	30.1	29.4	30	30.7	30.1	35.9
Chickpeas (g)	29.6	29.6	30.1	30.6	30.2	30.1
Potatoes (g)	0	0	0	0	4	103.4
Meat (veal) (g)	135.3	117.4	89.4	91.4	89.6	103.7
Olive oil (ml)	9.6	8.8	10.4	10.2	9.6	11.7
Wine (ml)	0	0	53.3	52.6	0	21.6
Milk (ml)	0	0	31	77	117.5	251.2
Chocolate (g)	0	0.1	0.7	25	22.6	30.2

Source: see Table 1. ADPV, sección Hospital, II.1.14, V-2.3/C-15, *Clasificación del personal que se calcula atenderá este establecimiento de 1899 a 1900 […]*, 1900. ADPV, sección Hospital, II.2.3, V-7.6/3, 6, 7, 13, 14, *libros de la vaquería*; Base de Datos Española de Composición de Alimentos (www.bedca.net/bdpub/index.php (accessed on 15 September 2021)).

**Table 3:** Daily intake of energy and macronutrients of foundlings in the Hospital General de Valencia, 1852–1931.

Product	1852–1854	1866–1868	1899–1901	1909–1911	1921–1923	1929–1931
Energy (kcal)						
Brown bread (g)	546 (37)	673 (44)	696 (44)	727 (41)	0	0
White bread (g)	289 (20)	283 (18)	275 (18)	287 (16)	1007 (58)	1207(54)
Rice (g)	117 (8)	114 (7)	116 (7)	119 (7)	117 (7)	139 (6)
Chickpeas (g)	100 (7)	99 (6)	101 (6)	103 (6)	101 (6)	101 (5)
Potatoes (g)	0	0	0	0	3 (0)	76 (3)
Meat (veal) (g)	345 (23)	299 (19)	228 (15)	233 (13)	229 (13)	264 (12)
Olive oil (ml)	85 (6)	78 (5)	92 (6)	91 (5)	85 (5)	104 (5)
Wine (ml)	0	0	38 (2)	37 (2)	0	15 (1)
Milk (ml)	0	0	20 (1)	50 (3)	76 (4)	163 (7)
Chocolate (g)	0	1 (0)	3 (0)	133 (7)	120 (7)	161 (7)
Total energy	1481	1548	1571	1780	1738	2230
Macronutrients (g)						
Proteins ^1^	37.2	37.2	34.2	37.8	38.3	49.1
Fats	45.7	42	39.2	48.8	48.5	62.5
Carbohydrates	204	227.3	232.9	259.6	258.3	328.7

Notes: The values in parentheses indicate the percentage that the caloric contribution of that food supposes with respect to the total of the daily energy intake. ^1^ Maximum biological value proteins have been estimated based on data provided by FAO [34]. Source: see Table 1 and Table 2.

**Table 4:** Contribution of energy and nutrients with respect to the Recommended Dietary Allowances (RDA = 100) in the average daily diet of foundlings in the Hospital General de Valencia, 1852–1931.

Product	1852–1854	1866–1868	1899–1901	1909–1911	1921–1923	1929–1931
Energy	92	96	97	110	107	137
Proteins	129	129	121	129	131	168
			Vitamins			
Vitamin A	2	2	6	13	19	37
Vitamin D	0	0	0	0	0	1
Vitamin E	35	33	36	38	37	45
Vitamin C	2	2	3	4	7	44
Thiamine	82	88	82	89	89	124
Riboflavin	50	49	51	65	71	109
Niacin	164	160	145	153	153	199
Vitamin B6	68	67	61	65	65	110
Folic acid	97	103	104	109	109	137
Vitamin B12	104	90	79	91	98	141
			Minerals			
Calcium	36	40	47	59	65	98
Phosphorus	136	138	135	153	157	212
Magnesium	81	87	90	109	106	143
Iron	126	131	133	146	138	173
Zinc	72	69	62	69	69	88
Iodine	31	33	37	47	52	80
Selenium	459	518	522	548	543	666

Source: see Table 1, Table 2 and Table 3; Recommended Dietary Allowance (RDA): [19]; Population structure data in the Spanish province of Valencia, 1877: [48]; 1900: [48]; 1910: [48]; 1920: [49]; 1930: [50].
